# Comparing blastocyst quality and live birth rates of intravaginal culture using INVOcell™ to traditional in vitro incubation in a randomized open-label prospective controlled trial

**DOI:** 10.1007/s10815-016-0661-0

**Published:** 2016-02-03

**Authors:** Kevin J. Doody, E. Jason Broome, Kathleen M. Doody

**Affiliations:** Center for Assisted Reproduction, 1701 Park Place Ave, Bedford, TX 76022 USA; Kelowna Regional Fertility Centre, Kelowna, BC Canada

**Keywords:** Assisted reproductive technology, Intravaginal culture, Mild stimulation, Minimal monitoring, In vitro fertilization, IVC, IVF, INVO, INVOcell

## Abstract

**Purpose:**

The purpose of this study is to to compare the efficacy of intravaginal culture (IVC) of embryos in INVOcell™ (INVO Bioscience, MA, USA) to traditional in vitro fertilization (IVF) incubators in a laboratory setting using a mild pre-determined stimulation regimen based solely on anti-mullerian hormone (AMH) and body weight with minimal ultrasound monitoring. The primary endpoint examined was total quality blastocysts expressed as a percentage of total oocytes placed in incubation. Secondary endpoints included percentage of quality blastocysts transferred, pregnancy, and live birth rates.

**Methods:**

In this prospective randomized open-label controlled single-center study, 40 women aged <38 years of age with a body mass index (BMI) of <36 and an AMH of 1–3 ng/mL were randomized prior to trigger to receive either IVC or IVF. Controlled ovarian stimulation was administered with human menopausal gonadotropin (hMG) in a fixed gonadotropin-releasing hormone (GnRH) agonist cycle based solely on AMH and body weight. A single ultrasound-monitoring visit was performed on the 10th day of stimulation. One or two embryos were transferred following 5 days of culture.

**Results:**

IVF produced a greater percentage of total quality embryos as compared to IVC (50.6 vs. 30.7 %, *p* = 0.0007, respectively). There was no significant difference between in IVF and IVC in the percentage of quality blastocysts transferred (97.5 vs. 84.9 %, *p* = 0.09) or live birth rate (60 % IVF, 55 % IVC).

**Conclusions:**

IVF was shown to be superior to IVC in creating quality blastocysts. However, both IVF and IVC produced identical blastocysts for transfer resulting in similar live birth rates. IVC using INVOcell™ is effective and may broaden access to fertility care in selected patient populations by ameliorating the need for a traditional IVF laboratory setting. Further studies will help elucidate the potential physiological, psychological, geographic, and financial impact of IVC on the delivery of fertility care.

## Introduction

The process of in vitro fertilization (IVF) where oocytes and sperm are incubated in a laboratory setting is necessary to achieve pregnancy for many patients with infertility. Unfortunately, the expense and overall burden of IVF significantly restricts access to assisted reproductive technologies (ART) [1]. The modern IVF laboratory requires expensive air filtration systems [2], as the embryo has no lung, kidney, or liver to filter air contaminants including volatile organic compounds. Incubators require alarm systems, daily quality control checks, and 24/7 monitoring. In addition, if intracytoplasmic sperm injection (ICSI) or similar micromanipulation is offered, highly trained embryologists with specialized skills enabling those procedures must be available. As a result, access to fertility programs offering IVF is generally restricted from a geographic perspective to large urban centers and from a financial perspective to those who can afford this treatment.

Intravaginal culture (IVC) was proposed nearly 30 years ago as a means to reduce the overall burden and increase access to reproductive care [3]. The IVC technique places oocytes and sperm into a gas permeable culture device (INVOcell™, INVO Bioscience, MA, USA), which is then inserted in the vaginal cavity for incubation allowing fertilization and embryo development to occur [4].

IVC therefore has the potential to remove the need for a sophisticated and costly IVF laboratory as well as reducing overall embryologist intervention. The associated reduced capital costs and operating expenses may improve affordability and access to ART services.

Two prospective non-randomized studies and one randomized investigation have examined the efficacy of IVC using INVOcell™ which augmented early studies utilizing prototype devices [5–9]. Lucena and colleagues employing a mild stimulation protocol found that in 125 cycles, IVC yielded a cleavage rate of 63 % and an ongoing pregnancy rate of 40 % [10]. The second prospective non-randomized study found that IVC/ICSI produced a cleavage rate of 79 % and a live birth rate of 53 %. This was found similar to an internal matched IVF/ICSI control group of 74 cycles, which generated a cleavage rate of 76 % and a live birth rate 58 % [11]. The third study examined sister oocytes from 10 women who were randomized to receive either IVC or IVF. Cleavage rates were found to be similar for both IVC (97 %) and IVF (93 %); however, IVC yielded a significantly lower total number of embryos suitable for transfer. The study was limited in that only IVC embryos were transferred and thus comparative pregnancy rates were not possible. The investigators found IVC produced a 30% clinical pregnancy rate [12].

The effectiveness of IVC to reduce the burden of fertility care can only be determined once its efficacy is compared directly to the traditional incubators used in IVF in a randomized controlled fashion. This randomized prospective open-label trial is the first to compare the blastocyst quality and resultant live birth rates of IVC to IVF. The primary endpoint examined was the total number of day 5 embryos of “good quality” defined as 2BB or greater (expansion of embryos 2, 3, 4, 5, or 6 and inner cell mass and trophectoderm grades of A or B) or higher using a modified Gardner Grading System [13] expressed as a percentage of total number of oocytes placed in incubation. Secondary endpoints were also examined and included percentage of quality embryos transferred and live birth rates.

## Methods

### Study setting

The study was carried out at C.A.R.E., Bedford, TX, USA. The study was a prospective randomized open-label trial. Ethics approval was obtained from an external Research Ethics Board (IRB Services, www.irbservices.com).

### Study population

From November 2012 until December 2013, a total of 40 infertile couples were enrolled into the study. Women presenting to the clinic between the ages of 18 and 38 years, who had failed to conceive after 1 year of unprotected intercourse, had a normal recent uterine cavity evaluation, and in which IVF was to be the next treatment, were invited to participate in the study. Male partners were required to have normal or only mildly abnormal semen parameters or better. Couples with a severely abnormal semen analysis (less than five million progressively motile sperm in the ejaculate) were excluded. Exclusion criteria included chronic illness, vaginal inflammation, infection, uterine anatomic abnormalities, or allergy to plastics. Other exclusion factors included severe endometriosis, untreated hydrosalpinx, BMI greater than or equal to 36, use of donor sperm or eggs, low ovarian reserve (AMH < 1 ng/ml), polycystic ovaries, prior history of ovarian hyperstimulation, inability to wear a diaphragm, smokers, drug or alcohol abuse, and two or more failed previous IVF cycles or an IVF cycle with where fertilization did not occur.

### Stimulation and monitoring

Oral contraceptives were used to program the cycle and overlapped with leuprolide acetate 1 mg/day. Leuprolide acetate was continued for 7–14 days and then decreased to 0.5 mg/day prior to stimulation. Sonogram was used to confirm absence of follicular cysts prior to starting leuprolide and prior to initiation of human menopausal gonadotropin (hMG). hMG (Ferring, NJ, USA) was used at a starting dose of 150 or 225 IU per day beginning on a Saturday such that monitoring, egg retrieval, and embryo transfer on weekends could be predictably avoided. The gonadotropin dose was either maintained or “stepped down” to 150 IU after one or more days, taking into account the AMH and body weight. A single sonogram-monitoring visit performed on the 10th day of stimulation. By study design, hCG trigger was limited to only three possible days. The decision to trigger on stimulation days 10, 11, or 12 (Monday, Tuesday, or Wednesday) was based on measurements of follicle size on day 10. Egg retrieval (scheduled for a Wednesday, Thursday, or Friday) was performed 36 h following trigger.

### Patient randomization

Patients were randomized on day 10 following their sonogram to receive either IVC or IVF with a single or double embryo transfer on day 5. Randomization was done through lottery system where 20 IVF and 20 IVC cards were placed in an opaque envelope and a single card was drawn by the clinical nurse coordinator for each patient. Forty-four patients were enrolled and four patients declined to move forward for personal reasons during the consenting process. The remaining 40 patients, 20 in each arm represented complete enrollment as per study protocol, and all 40 completed the study. Because randomization was done following the day 10 sonogram, prior to retrieval, neither the physician nor embryologist was blinded with regard to oocyte assessment and blastocyst scoring.

### Oocyte culture

Up to 10 oocytes (as per the Instructions For Use included with the device) were co-incubated with sperm for 2 to 4 hours. These oocytes were then cultured in traditional tri-gas incubators or the vaginal culture device INVOcell™ (INVO Bioscience, MA, USA) for 5 days in continuous culture media and serum substitute supplement (both Irvine Scientific, Santa Ana, CA, USA) and in the case of IVC culture 0.3-mL oil for embryo culture (Irvine Scientific, Santa Ana, CA, USA). If more than 10 oocytes were obtained, the extra oocytes were vitrified or discarded. The study protocol was designed to minimize the risk of ovarian hyperstimulation syndrome (OHSS) such that all embryos would be vitrified and embryo transfer would be deferred if more than 20 oocytes were obtained.

### Embryo transfer

After 5 days of continuous culture, the embryos were removed from the INVOcell™ or traditional incubators and carefully identified and scored according to the Gardner Scoring System. One or two selected embryos were loaded and transferred into the uterus using a standard embryo transfer catheter and ultrasound guidance. The decision to transfer either one or two embryos was made by the patient after consideration of patient age, embryo quality, and the availability of additional embryos for cryopreservation in accordance with the guidelines published by the American Society of Reproductive Medicine [14]. Multiple embryologists were utilized for both IVF and IVC cycles, and care was taken to ensure concordant scoring via ongoing internal quality control measures.

### Pregnancy assessment

All patients were monitored for chemical pregnancy nine or more days after embryo transfer and sonographically assessed after 6 weeks gestation for fetal heart. Patients were contacted following 9 months gestation to collect the live birth data.

### Outcome measures and statistical analysis

The primary endpoint was total quality blastocysts expressed as percentage of oocytes incubated. Secondary outcome measures included percentage of quality embryos transferred as well as pregnancy and live birth rates. Statistical analysis was performed using *Z* test 2 population proportions (comparative embryo data) and Student’s *t* test (comparative patient data, with the exception of embryos transferred which was not normally distributed and so a Mann-Whitney *U* test was deployed). *P* < 0.05 was considered statistically significant. The sample size of total blastocysts required per treatment group was calculated to be 84 assuming an error rate of 0.05%, a power of 0.90, and an effect size of 0.5.

## Results

Forty (40) stimulation cycles were performed, and a total of 314 oocytes were collected. One hundred percent of cycles yielded 1 or more oocyte, and all cycles went to retrieval. One hundred and twenty seven (127) oocytes were placed in the vaginal culture device INVOcell™, and 156 oocytes were incubated in a traditional IVF culture system. Eighty-seven (87) of the 127 vaginally cultured oocytes (69%) were noted to have developed beyond the one cell stage. In contrast, the oocytes cultured in the conventional incubators had an 88% cleavage rate, producing 115 embryos from 131 oocytes. Additional comparative patient, oocyte, and embryo data is presented in Table 1.

**Table 1:** Patient comparison IVC (*n* = 20) vs. IVC (*n* = 20)

	IVC	IVF	*P* value
Age (years)	33.1/34.0 (26–38)	32.3/32.0 (26–38)	0.45
AMH (ng/mL)	1.87/1.86 (1.07–2.90)	1.84/1.73 (1.08–2.91)	0.87
Weight (lbs)	148/154.5 (100–184)	161/158.5 (121–207)	0.12
Gonadotropin usage (I.U.)	1845/1912.5 (1350–2475)	1882/1912.5 (1425–2475)	0.71
Oocytes retrieved	6.8/5.0 (3–15)	9.0/8.0 (1–18)	0.06
Eggs cleaved	4.1/4.0 (1–9)	5.8/6.0 (1–10)	0.03
Embryos transferred	1.65/2.0 (1, 2)	1.80/2.0 (1, 2)	0.42

Mean and median results are presented respectively with range in parenthesis

**Fig. 1:**
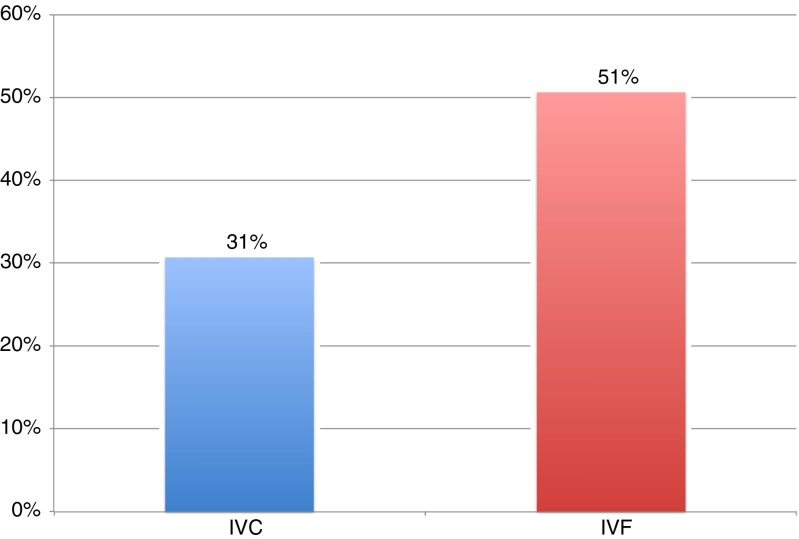
Total number of embryos greater than or equal to 2BB as percentage of the total number of oocytes incubated (IVC = 127, IVF = 156) *p* = 0.0007

Thirty-nine (39) embryos generated from the 127 vaginally cultured oocytes (31%) were graded greater than or equal to 2BB based on the Gardner Scoring System. In contrast, 79 of the 156 (51%) IVF cultured embryos were graded greater than or equal to 2BB (Fig. 1). The difference between IVC and IVF was found to be statistically significant (*p* = 0.0007).

**Fig. 2:**
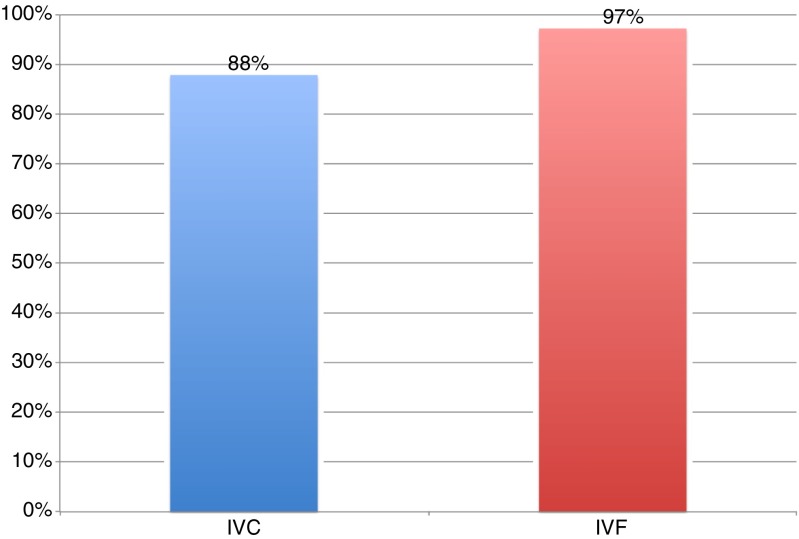
Embryos greater than or equal to 2BB as a percentage of the total number of embryos transferred (IVC = 33, IVF = 36) *p* = 0.09

**Fig. 3:**
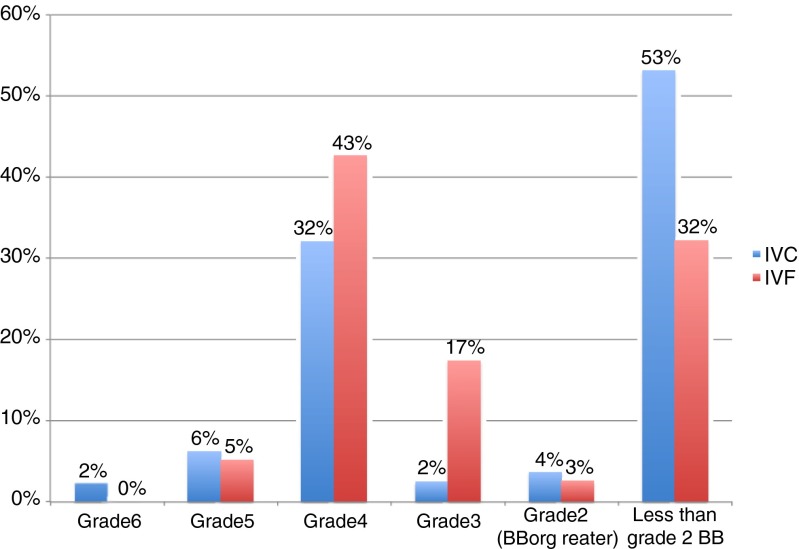
Cleaved embryos distributed by Gardner Grading System for IVC and IVF (IVC = 81, IVF = 115)

When examining those embryos that were transferred, however, the statistically significant difference was not observed between IVC and IVF embryos with 87.9 and 97.2 % scoring greater than or equal to 2BB, respectively (*p* = 0.09, Fig. 2). Figure 3 illustrates the distribution of embryos’ grades across both IVC and IVF. Only IVC generated the highest graded 6AA embryos in 2 cycles. In grades 4 and 3, IVF produced greater numbers of embryos relative to IVC.

**Fig. 4:**
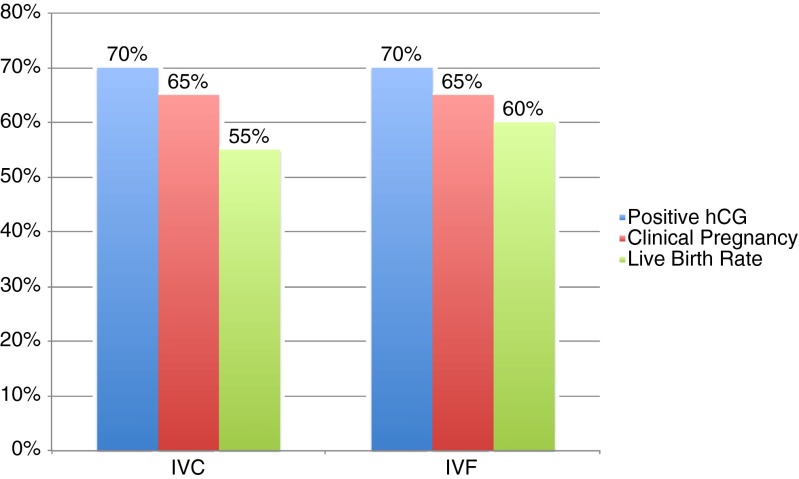
Pregnancy rates for IVC (*n* = 20) and IVF (*n* = 20)

All 40 cycles went to embryo transfer with either a single or double embryo transfer performed. All cycles were assessed to a minimum of fetal heart confirmation on week 6 by sonogram. The 20 IVC cycles produced 14 biochemical pregnancies, 13 clinical pregnancies, and 11 pregnancies resulting in live birth(s). A total of 16 live born infants (5 sets of twins) were observed with an average birth weight of 5.84 lb. Similarly, the 20 IVF cycles produced 14 biochemical pregnancies, 13 clinical pregnancies, and 12 pregnancies resulting in live birth(s). A total of 15 live born infants (3 sets of twins) were observed with an average birth weight of 5.94 lb. Comparative pregnancy rates between IVC and IVF are presented in Fig. 4.

No cycle produced more than 18 oocytes. Thus, no cycle required “freeze-all” to reduce the risk of OHSS. Similarly, no patient experienced significant OHSS symptoms requiring additional office visits or intervention. Additionally, no patients reported losing INVOcell™ from the vaginal cavity. The most common reported adverse events captured in a post cycle questionnaire which was administered to the couples in the IVC arm were vaginal discharge (45 %), discomfort related to the device (15 %), device becoming dislodged requiring repositioning (15 %), itching (15 %), and spotting (10%).

## Discussion

The burden of reproductive health is often limited from a geographic, financial, and time perspective. Care has to a large degree been consolidated into substantial facilities in large urban centers providing ever increasingly costly IVF- and IVF-related procedures [15]. To reduce the burden of care ideally all three barriers: time, geographical constraints, and cost are adequately addressed. Recently, a simplified culture system was proposed which sought to reduce the cost of IVF by replacing a full laboratory with a so-called shoebox-sized environment. In validating the simplified approach, Van Blerkom and colleagues reported an implantation rate of 35 % and seven healthy live births [16]. Another simplified form of IVF, which also sought to replace the complex IVF laboratory, has been proposed which uses intravaginal culture (IVC) [4]. Specifically, a gas permeable medical device (INVOcell™, INVO Bioscience, MA, USA) containing culture media with spermatozoa and oocytes and placed in the vaginal cavity acts as the necessary pCO2/pO2 incubator [17], supplying the environment and temperature to facilitate fertilization and early embryo development. IVC procedures have been performed in a number of countries with pregnancy rates ranging from 20 to 59 % [3, 13, 15–18].

Despite the increasing adoption of IVC as a complementary approach to IVF, to date an adequate comparison to IVF has not yet been performed. The study presented here is the first to compare IVC to IVF in a randomized fashion and demonstrates that IVF is superior to IVC in generating day 5 embryos of quality greater than or equal to 2BB as per the Gardner Scoring System (50.6 vs. 30.7 %, *p* = 0.0007, respectively). However, when considering the quality of the embryos transferred, IVF and IVC had similar percentages of embryos graded 2BB or greater (97.2 and 87.9 %, respectively), resulting in near identical live birth rates (60 % for IVF and 55 % for IVC).

Utilizing the vaginal cavity to maintain temperature and provide the appropriate low oxygen and high CO_2_ environment has clear advantages in terms of reducing both the geographic restriction and cost as it ameliorates the need to build and maintain a full IVF laboratory significantly reducing the size and thus cost of a facility providing IVF services. Further optimal embryo development has been shown to be influenced by even minor changes in environmental conditions such as pH, temperature, and oxygen concentration [19]. IVC has the advantage of eliminating potential fluctuations in those parameters as the frequent assessments of embryo development have been shown to negatively affect embryo development [18]. While IVC does not allow monitoring or adjustments to either O_2_ or CO_2_ concentrations, it has been shown to maintain levels of relatively high CO_2_ levels and low pH by equilibration of the culture medium with intraepithelial vaginal gas even in metabolically active medium containing sperm and oocytes [20].

Artificial environments, which seek to mimic the in vivo environment, have been shown to improve embryo development [21]. IVC allows developing embryos to be exposed to these natural rhythms, which may positively enhance embryo development. It is important to note that this potential benefit needs to be balanced with an inability of the IVC process to allow the assessment of early fertilization development. It is interesting that only IVC, in two cycles, generated the highest quality 6AA embryo on day 5. Further utilization of IVC and refinement of protocols will aid in determining if natural body rhythms and associated microfluidic environment of IVC is potentially a superior incubating system to traditional IVF systems.

This study suggests that IVC using INVOcell™ is a viable alternative option for assisted reproduction. Additional studies validating IVC as a treatment option for infertile patients are required to fully elucidate the ideal patient profile and gain a better understanding of the cumulative pregnancy rate of IVC as compared to IVF as significant differences were noted between total quality embryos generated.
